# Design of a Digital LAMP Detection Platform Based on Droplet Microfluidic Technology

**DOI:** 10.3390/mi14051077

**Published:** 2023-05-19

**Authors:** Liying Jiang, Xianghao Lan, Linjiao Ren, Mingzhu Yang, Bo Wei, Yang Wang

**Affiliations:** 1 School of Electrical and Information Engineering, Zhengzhou University of Light Industry, Zhengzhou 450002, China; 2 Academy for Quantum Science and Technology, Zhengzhou University of Light Industry, Zhengzhou 450002, China; 3 Beijing Research Institute of Mechanical Equipment, Beijing 100143, China; 4 Department of Thoracic Surgery, Beijing Tiantan Hospital, Capital Medical University, Beijing 100070, China; 5 Beijing Advanced Innovation Center for Biomedical Engineering, Key Laboratory for Biomechanics and Mechanobiology, School of Engineering Medicine, Beihang University, Beijing 100083, China

**Keywords:** microfluidic, droplet generation, digital-LAMP, high sensitivity

## Abstract

Loop-mediated isothermal amplification (LAMP) is a rapid and high-yield amplification technology for specific DNA or RNA molecules. In this study, we designed a digital loop-mediated isothermal amplification (digital-LAMP)-functioning microfluidic chip to achieve higher sensitivity for detection of nucleic acids. The chip could generate droplets and collect them, based on which we could perform Digital-LAMP. The reaction only took 40 min at a constant temperature of 63 °C. The chip enabled highly accurate quantitative detection, with the limit of detection (LOD) down to 10^2^ copies μL^−1^. For better performance while reducing the investment of money and time in chip structure iterations, we used COMSOL Multiphysics to simulate different droplet generation ways by including flow-focusing structure and T-junction structure. Moreover, the linear structure, serpentine structure, and spiral structure in the microfluidic chip were compared to study the fluid velocity and pressure distribution. The simulations provided a basis for chip structure design while facilitating chip structure optimization. The digital-LAMP-functioning chip proposed in the work provides a universal platform for analysis of viruses.

## 1. Introduction

Due to the low concentration of target nucleic acids in scenarios of disease diagnosis, food safety control, and environmental monitoring, nucleic acid testing (NAT) has been widely used owing to its high sensitivity and specificity [1,2,3]. At present, there are several nucleic acid amplification technologies, of which polymerase chain reaction (PCR) [4] is the most commonly used. In a typical PCR process, nucleic acid molecules are exponentially amplified with primers at 2–3 different temperatures. Such a “thermal cycle” reaction process requires accurate temperature control, resulting in high costs. To eliminate the thermal cycle, isothermal amplification methods have been developed, such as loop-mediated isothermal amplification (LAMP) [5], which reduces the need for complex and expensive temperature control modules. Moreover, the LAMP amplification method offers additional primers (usually 4–6) that can make the detection more specific, and its amplification process only takes 60 min or less [6,7,8,9,10,11]. Owing to these advantages, LAMP is widely used in nucleic acid detection. However, in some cases, the concentration of target nucleic acids is still too low to be detected by using the traditional LAMP amplification method. To achieve higher sensitivity, a digital loop-mediated isothermal amplification (digital-LAMP) [12,13] has been developed, which was expected to achieve higher precision, higher sensitivity, and absolute quantitative analysis of test samples. Offering the advantages of simple instrumentation and fast reaction speed, digital-LAMP provides a powerful and reliable analysis platform for the rapid diagnosis and prevention of diseases [14]. 

At present, digital-LAMP is often performed on droplet microfluidic chips [15]. Research on droplet microfluidic chips with different functions has gradually increased [16]. To generate fluid droplets suitable for digital-LAMP, the fluidic samples need to first be split into appropriate sizes within the droplet microfluidic chip, which requires a lot of optimization work on the chip structure. This costs considerable amounts of money and time. COMSOL Multiphysics was therefore widely used to build a model for simulation analysis, which greatly reduced the cost and time for chip optimization. Wong et al. used COMSOL Multiphysics to construct a T-junction structure and flow-focusing structure that can generate droplets and explored the factors that affect the effective diameter of droplets [17]. Fang et al. used COMSOL Multiphysics to construct mixed structures that can mix different fluids and study the mixing efficiency of different fluids [18]. These simulation results based on COMSOL Multiphysics provide important references for chip design. However, most of the existing studies that applied COMSOL Multiphysics only carried out simulation analysis rather than real experiments. 

Considering the fact that the real performance of the designed microfluidic chip was actually determined by real experiments, in this work, we used the simulation results of COMSOL Multiphysics to guide the actual chip fabrication. We first used COMSOL Multiphysics to build models of flow-focusing structure and T-junction structure that can form droplets. Through the comparison of the simulation results of different droplet generation structures, it was found that the size range and uniformity of the flow-focusing structure was better. We then simulated various microflow channel structures, including linear, serpentine, and spiral, for studying the velocity and pressure distribution. Through comparison, it was found that the velocity and pressure distribution of the serpentine structure were better than those of the linear structure after fluid injection. Moreover, the spiral structure has certain advantages that enable the formed droplets to be evenly laid in the collection chamber. The simulation results were used to guide the optimization and real fabrication of the microfluidic chip. The digital-LAMP technique was then integrated into the chip with microfluidic droplet generation function to realize high sensitivity and fast prediction of SARS-CoV-2 concentration, achieving a limit-of-detection (LOD) down to 10^2^ copies μL^−1^. Based on the digital isothermal amplification method, the chip can fulfill the test requirements even in rural areas and laboratories with insufficient experimental equipment. Moreover, the chip can provide an experimental platform and a reference basis for the detection of other virus samples.

## 2. Experimental

### 2.1. Materials and Reagents

The 2× WarmStart LAMP Kit (E1700S) was purchased from New England Biolabs (Beijing, China). The primers were synthesized by Sangon (Shanghai, China). The sequences of all the primers are listed in Table 1. Polydimethylsiloxane (PDMS) was purchased from Dow Corning (New York, NY, USA). In vitro transcribed (IVT) RNA was transcribed from a plasmid that contained the sequences of the N gene with a MEGAscript T7 transcription kit (Thermo Fisher Scientific, Waltham, MA, USA). Fluorinert HFE-7500 fluorocarbon oil was purchased from 3M (Beijing, China).

### 2.2. Geometric Structure Design of the Chip

**Figure 1:**
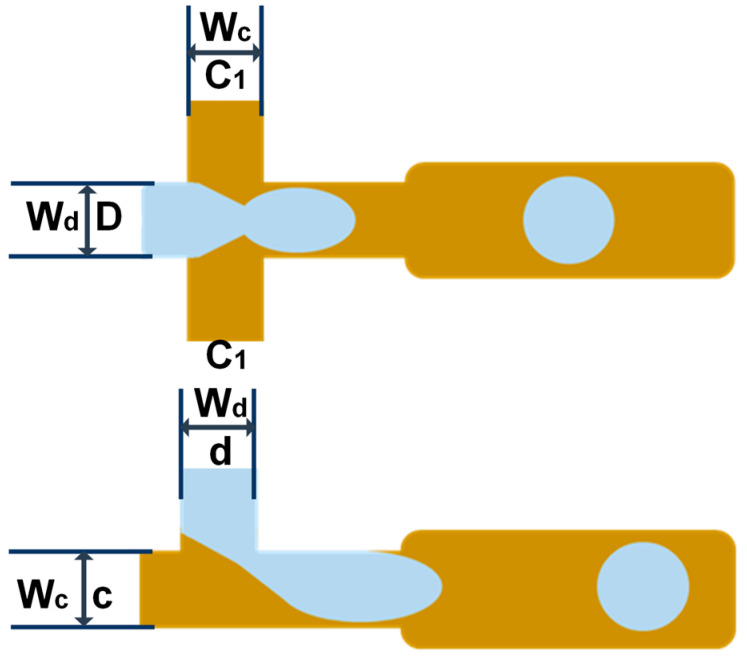
Flow-focusing structure and T-junction structure geometry.

**Figure 2:**
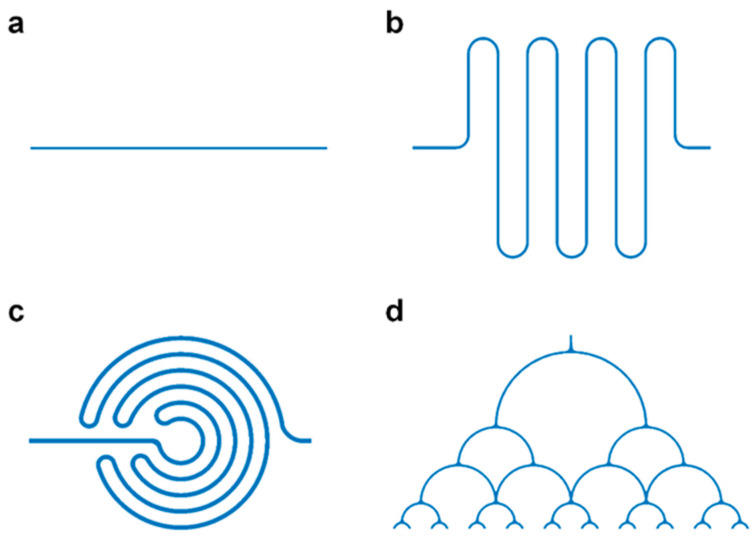
Various microfluidic channels: (**a**) linear structure; (**b**) serpentine structure; (**c**) spiral structure; (**d**) splitting structure.

Figure 1 shows the flow-focusing and T-junction geometric structures for droplet generation, in which the continuous phase-width Wc and the dispersed phase-width Wd were both set to 50 μm. We also used COMSOL to establish various microflow channel structures for analysis. Figure 2 shows the linear structure, serpentine structure, spiral structure, and splitting structure of fluid flow. The channel widths of the linear structure and the serpentine structure were both 50 μm. The fluid velocity and pressure distribution were studied and compared before droplet formation. Subsequently, the formed droplets flew into the spiral structure and splitting structure, whose widths were both 100 μm. 

### 2.3. Governing Equations of COMSOL

The geometry of the microchannel was imported into COMSOL Multiphysics, a numerical simulation software for implementing finite element method analysis. There were two control equations used as calculation tools in the simulation. The level set method was used to study the effect of liquid droplets generated by the flow-focusing structure and T-junction structure on the fluid in different microchannels. In level set, the liquid flow rate of two-phase flow was relatively low through the microscale channels. Here, the two phases can be regarded as homogeneous and incompressible fluids. Under the condition of producing micron-sized droplets, the influence of fluid gravity on the flow rate and flow velocity was negligible. Therefore, to study the dynamics of droplet deformation, it was necessary to numerically solve the Navier—Stokes equation with simplifications as follows:

∇ · u=0

$$\frac{\rho \partial u}{\partial t}+\rho \nabla \;\cdot \;\left(uu\right)=-\nabla p+\nabla \cdot \left[\;\mu \left(\nabla u+\nabla u^{T}\right)\right]+F$$

$$\frac{\partial \emptyset }{\partial t}+u\;\cdot \;\nabla \emptyset =\gamma \nabla \;\cdot \;\left[\varepsilon \nabla \emptyset -\emptyset \left(1-\emptyset \right)\frac{\nabla \emptyset }{\left|\nabla \emptyset \right|}\right]$$

where *u* is the fluid velocity, ***p*** is the fluid pressure, ***ρ*** is the dynamic density of the fluid, ***μ*** is the dynamic viscosity coefficient, ***F*** is the surface tension, ∅ represents the level set function, and ***γ*** and ***ε*** are numerical stabilization parameters. ***ρ*** and ***μ*** can be determined by the following equations:

$$\rho =\rho_{c}+\left(\rho_{c}-\rho_{d}\right)\emptyset$$

$$\mu =\mu_{c}+\left(\mu_{c}-\mu_{d}\right)\emptyset$$

where ***ρ*_c_**, ***ρ*_d_**, ***μ*_c_**, ***μ*_d_** represent the density and viscosity of the continuous phase and dispersed phase, respectively, and the effective diameter ***d*_eff_** of microdroplets was calculated as follows:

$$d_{eff}=2\;\cdot \;\sqrt[{3}]{\frac{3}{4\pi }\int \left(\emptyset >0.5\right)d\Omega }$$

### 2.4. Parameter Setting

The simulation used Fluorinert HFE-7500 fluorocarbon oil as the continuous phase fluid, setting its density at 1.164 × 10^3^ kg m^−3^ and dynamic visibility at 1.12 × 10^−2^ Pa·s. The simulation used water as the dispersed-phase fluid, setting its density at 1 × 10^3^ kg m^−3^, dynamic visibility at 1.01 × 10^−2^ Pa·s, surface tension at 5 × 10^−3^ N m^−1^, and contact angle θ at 3/(4π), which is the included angle between the fluid interface and the wall at the contact point between the fluid interface and the solid wall. It was assumed that the initial state of the channel was to be filled with continuous-phase fluid, and the continuous-phase fluid and dispersed-phase fluid would inflow from the inlet at flow rates *u*_c_ and *u*_d_, respectively. In the simulation process of droplet generation, we set the dispersed-phase flow rate *u*_d_ as 0.005 m s^−1^, and the continuous-phase flow rate *u*_c_ as 0.005 m s^−1^, 0.01 m s^−1^, 0.015 m s^−1^, 0.02 m s^−1^, 0.025 m s^−1^, 0.035 m s^−1^, 0.035 m s^−1^, 0.04 m s^−1^, 0.045 m s^−1^, and 0.045 m s^−1^, respectively, corresponding the continuous- and dispersed-phase flow rate ratios (*u*_c/_*u*_d_) from 1 to 10.

### 2.5. Manufacturing Microfluidic Devices

To manufacture microfluidic chips, a master mold was first formed using a standard SU-8 lithography process. In short, after the silicon wafer cleaning process, the silicon wafer was coated with a 50 μm thick SU 8-2050 layer, and then the composite unit was patterned with UV exposure through a photolithographic mask. Then, we carried out a two-step post-exposure baking process, washed the remaining photoresist with acetone and isopropanol, and completed the manufacturing of the master mold. Briefly, a polydimethylsiloxane (PDMS) elastomer precursor and curing agent were first mixed at a weight ratio of 10:1, and then, after degassing in a vacuum dryer, the mixture was poured into an SU-8 pattern mold. The mold was heated at 80 °C for 120 min (to yield incompletely cured PDMS), and then the reverse microstructure was formed by a mechanical demolding process. Then, the glass cover was bonded to the top of the microfluidic structure layer by oxygen plasma treatment to complete manufacturing of the liquid droplet microfluidic chip.

### 2.6. Digital-LAMP Detects SARS-CoV-2

**Figure 3:**
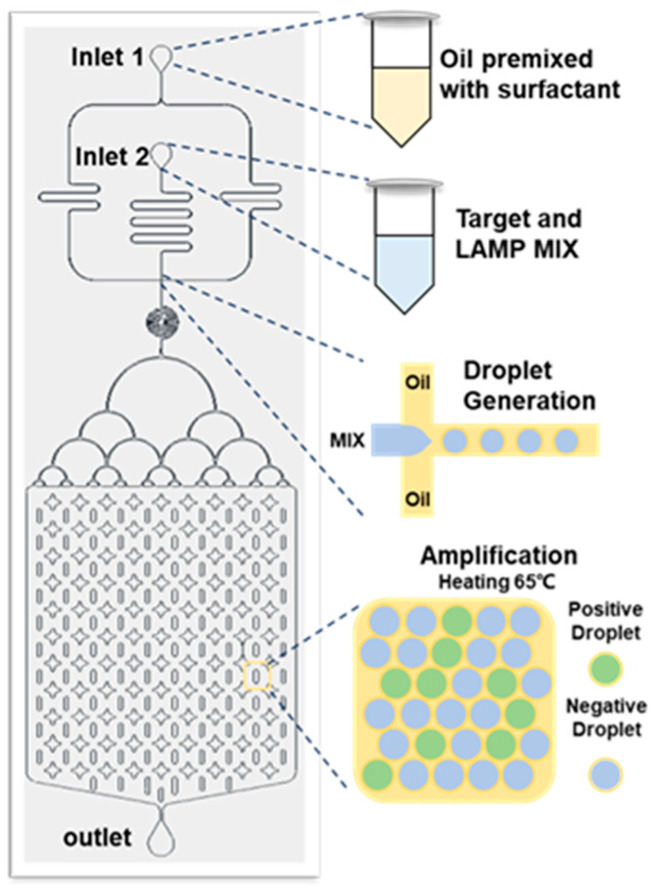
Detection of SARS-CoV-2 by droplet generation chip combined with digital-LAMP.

Before testing, the LAMP mixture solution was prepared. The LAMP mixture solution contained 5 μL WarmStart LAMP 2 × Master Mix, 1 μL 10 × LAMP fluorescent dye, 1 μL 10 × primers, 2 μL nuclease-free water, 1 μL IVT samples of SARS-CoV-2, and 10 × primer mixture, including a pair of outer primers (F3 and B3, 2 μM), a pair of inner primers (FIP and BIP, 16 μM), and a pair of loop primers (LF and LB, 8 μM). The sequences of all the primers are listed in Table 1. After all the solutions were added, vortex oscillation was used to uniformly mix the solution. The target sequences with different concentrations were then mixed with the LAMP mixture and connected to the droplet microfluidic dispersion phase inlet (Inlet 2) through the injection pump, and the fluorinated oil containing 2% surfactant was connected to the continuous-phase inlet (Inlet 1) of the microfluidic droplet generation chip through the injection pump. The LAMP mixture formed water-in-oil droplets on the chip through the formation process of the flow-focusing structure. The generated droplets were collected in the collection chamber of the droplet microfluidic chip and placed in a water bath at 63 °C to heat the chip for 40 min. Each droplet could be regarded as a separate LAMP amplification reaction system and could achieve exponential amplification of target RNA by the LAMP amplification method. The fluorescence intensity of the positive droplets with target RNA was high and could easily be observed under a fluorescence microscope under the dark-field condition. The control droplets (without target RNA) had weak fluorescence intensity and could not be observed. The statistical analysis of the number of fluorescent-positive droplets. In accordance with the above method, we performed the digital-LAMP test on the NTC samples and IVT samples with concentrations of 1 copy μL^−1^–10^5^ copies μL^−1^ and analyzed the sensitivity and detection limit of the test in Figure 3.

## 3. Results

**Figure 4:**
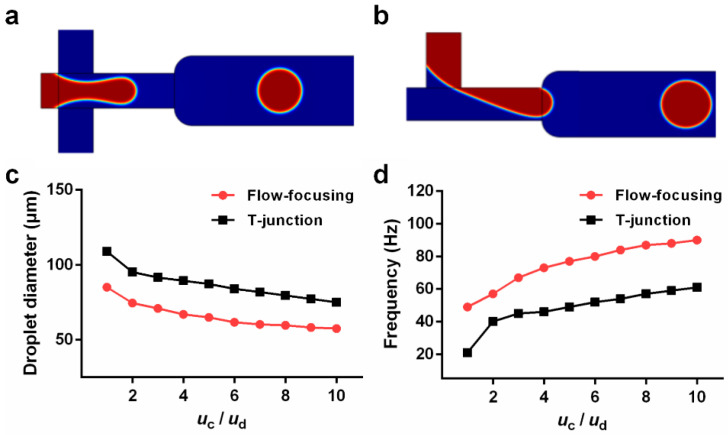
COMSOL-built model to generate droplets: (**a**) flow-focusing structure, (**b**) T-junction structure, (**c**) relationship between the diameter of the generated droplet and the flow rate ratio, and (**d**) relationship between the frequency of generating droplets and the flow rate ratio.

**Figure 5:**
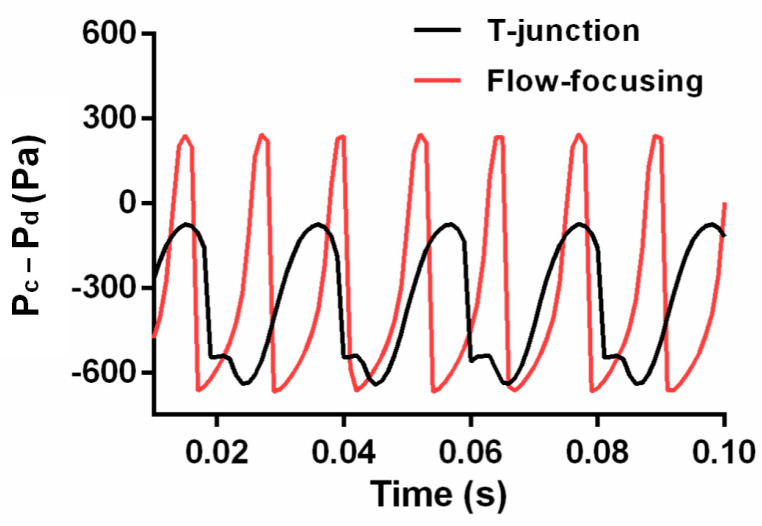
The pressure difference between the continuous phase and the dispersed phase when *u*_c_/*u*_d_ was 5.

Different structures of the droplet microfluidic chip produced different droplet effects. To ensure the stable generation of droplets, this paper mainly analyzes the diameters of the generated droplets and the frequency of droplet generation. For realistic biological detection, the volume of the biological sample was fixed. Considering the constant sample volume, the smaller the diameter of droplets generated, the more droplets could be generated, and the higher the accuracy of the detection. At the same time, the frequency of droplet generation would also affect the detection efficiency. Figure 4a,b shows the droplet formation of the flow-focusing structure and the T-junction structure. When maintaining the same flow rate ratio of continuous phase (oil phase) to dispersed phase (water phase), the velocity of the dispersed phase (*u*_d_) was set at 0.005 m s^−1^, and the velocity ratio of the continuous phase (*u*_c_) was set at 0.005 m s^−1^, 0.01 m s^−1^, 0.015 m s^−1^, 0.02 m s^−1^, 0.025 m s^−1^, 0.03 m s^−1^, 0.035 m s^−1^, 0.045 m s^−1^, 0.045 m s^−1^, 0.045 m s^−1^, and 0.045 m s^−1^, and the corresponding flow-rate ratio of the continuous phase to the dispersed phase (*u*_c_/*u*_d_) was from 1 to 10. The effective diameter of the droplets generated by the two types of droplet formation structures shown in Figure 4c would increase with increasing *u*_c_/*u*_d_, and the droplet generation frequency shown in Figure 4d would increase with increasing *u*_c_/*u*_d_. However, under the same *u*_c_/*u*_d_ conditions, the effective diameter and the frequency of droplet generation of the T-junction structure were less than those of the flow-focusing structure. Paramanantham et al. (2022) elaborated on the principle of droplet generation in their study of the T-junction structure. The interphase pressure difference near the intersection of the continuous phase and the dispersed phase was obtained according to the pressure gradient in the dispersed phase. Due to the pressure difference and shear force near the intersection, the dispersed phase lost its thickness, the interfacial tension of the continuous phase and the dispersed phase was insufficient to withstand the shear force and pressure difference, and the dispersed phase broke to form droplets [19]. The droplets were separated and flowed into the main horizontal channel. The shear force was affected by the dynamic viscosity and two-phase flow rate. Therefore, with the increase in the flow rate ratio *u*_c_/*u*_d_, the shear force increased, the droplet formation was faster, and the effective diameter was smaller. As shown in Figure 5, the pressure difference (P_c_ − P_d_) between the continuous phase and the dispersed phase was analyzed under the same flow rate ratio of *u*_c_/*u*_d_ = 5 for the flow-focusing structure and the T-junction structure. The pressure difference between the two phases of the flow-focused structure was greater than that of the T-junction structure, which was more conducive to the generation of droplets.

**Figure 6:**
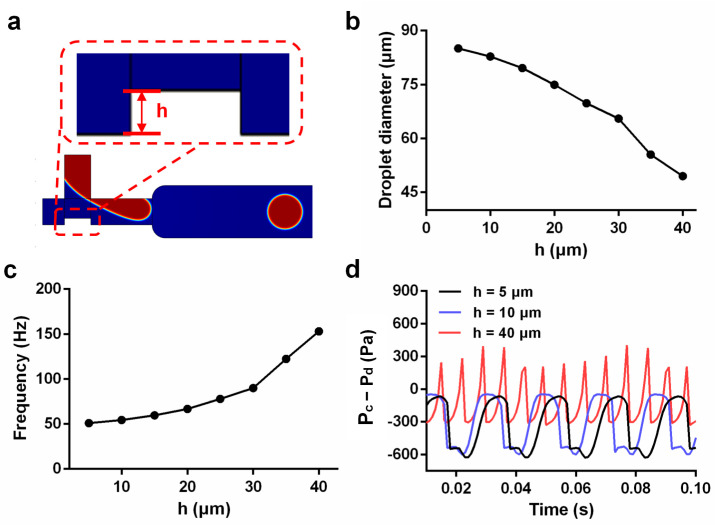
Simulation analyzed for the improvement of the T-junction structure: (**a**) optimization of the T-junction structure simulation diagram; (**b**) relationship between the height (h) and the diameter of the droplet in the optimized structure; (**c**) relationship between the height (h) and the frequency of droplet generation in the optimized structure; (**d**) change in pressure difference between the continuous phase and dispersed phase corresponding to the formation of liquid droplets at heights (h) of 5, 10, and 40 μm.

Based on the comparison, under the same conditions, the effect of droplet generation in the flow-focusing structure was significantly better than that of the T-junction structure. However, with only one continuous-phase passageway, the T-junction structure could greatly save oil. Thereafter, we tried to optimize the T-junction structure. Figure 6a shows a schematic diagram of the model structure for changing the width of the channel where the dispersed phase intersects with the continuous phase. At the same flow rate ratio of *u*_c_/*u*_d_ = 5, the effective diameter of the generated droplets shown in Figure 6b decreased with increasing height (h). Figure 6c shows the frequency of droplet generation increased as height (h) increased. Then, we analyzed the pressure difference between the two phases. Figure 6d shows the pressure difference (P_c_ − P_d_) changes between the continuous phase and the dispersed phase corresponding to the generation of droplets when the height h was 5 μm, 10 μm, and 40 μm. The pressure difference (P_c_ − P_d_) between the continuous phase and the dispersed phase at a height of 40 μm was significantly greater than those at 5 and 10 μm, and a height of 40 μm was more conducive to forming liquid droplets. However, when the step height rose to 35 μm, the width of the channel was only 15 μm, and this would increase the difficulty and cost of chip processing. Therefore, we chose the flow-focusing structure in the droplet microfluidic chip.

Through the comparisons between Figure 4, Figure 5 and Figure 6, we found that under the same conditions, the effective diameter of the droplets generated by the flow-focusing structure was smaller than that of the T-junction structure, in the meantime, with a higher frequency. This was attributed to the fact that two continuous-phase channels were included in the flow-focusing structure, leading to a higher difference of pressure between the continuous phase and the dispersed phase, which was more conducive to the formation of droplets. With the advantages of high generation efficiency and easy processing as well as a good droplet generation function, the flow-focusing structure was selected for further use.

**Figure 7:**
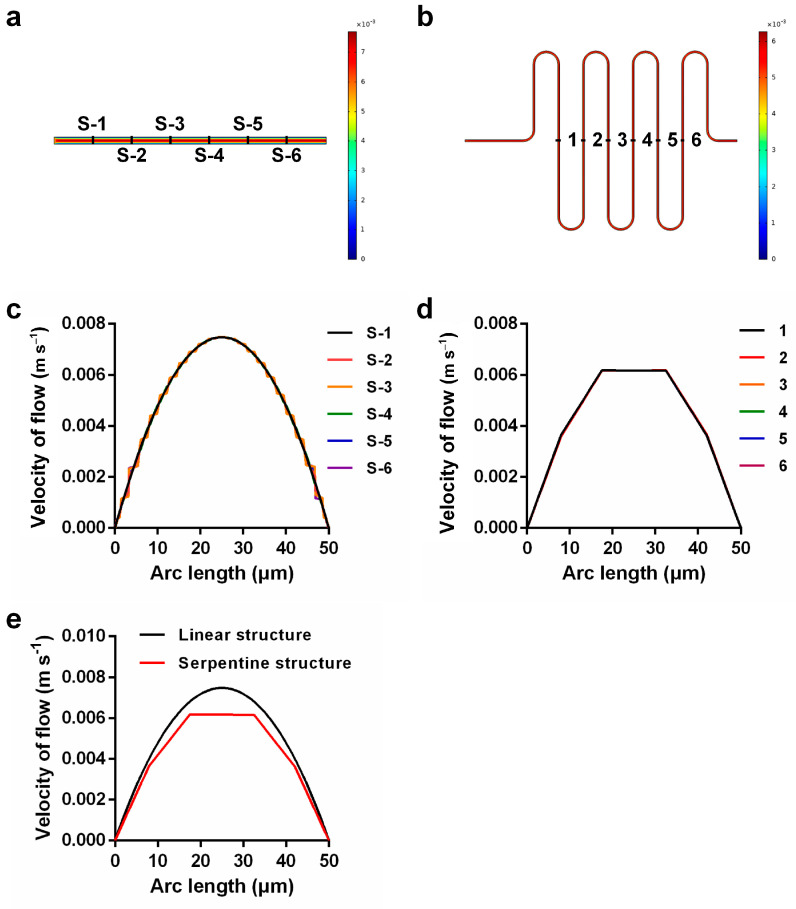
(**a**,**b**) Velocity distribution maps corresponding to linear and serpentine structures; (**c**) velocity of six uniformly selected positions in the linear structure; (**d**) velocity of six uniformly selected positions in the serpentine structure; (**e**) comparison of velocity at the exit of the two structures.

**Figure 8:**
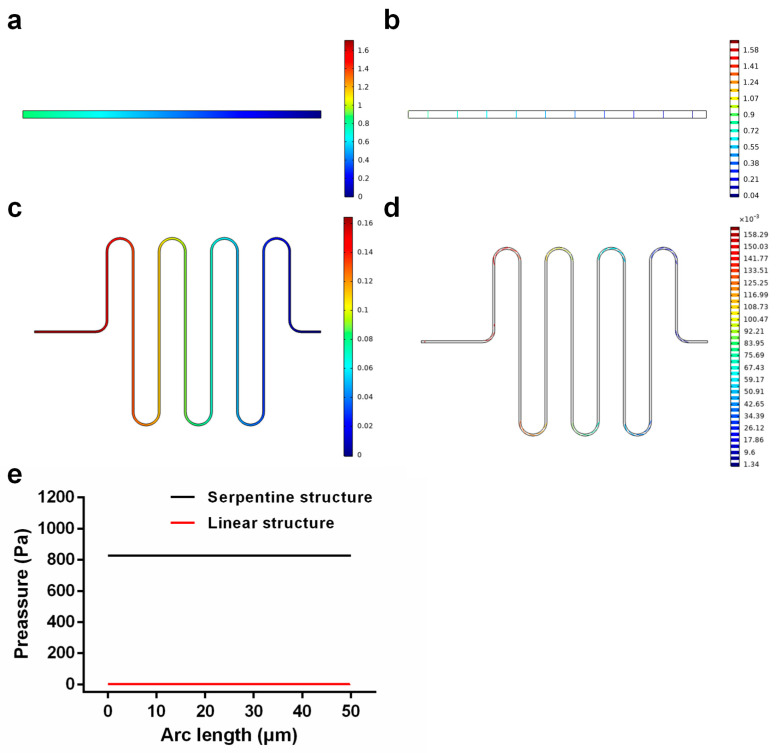
(**a**,**b**) Surface diagram and line diagram of pressure distribution in linear structures; (**c**,**d**) surface diagram and line diagram of pressure distribution in serpentine structure; (**e**) inlet pressure of serpentine structure and linear structure.

The velocity of flow through the microchannel was very fast. To prevent the injection fluid from flowing directly to the intersection of two phases while the initial velocity was still unstable, we used a serpentine structure instead of a linear structure to buffer the fast flow velocity. Figure 7a,b shows the velocity distribution diagram of the linear structure and serpentine structure, and Figure 7c,d shows the velocity distribution of six uniformly selected positions in the linear structure and serpentine structure. We found that the velocity distribution was uniform, the measure was consistent, and the velocity increased gradually from the wall to the center. However, the velocity at the center of the serpentine structure remained consistent, and this effect was better than the maximum velocity distribution at the center of the linear structure. In addition, the velocity of the center of the serpentine structure shown in Figure 7e was lower than that of the linear structure, indicating that the serpentine structure had a certain buffering effect. Then, we analyzed the pressure distribution of the two structures such that the pressure at the outlet was consistent. Figure 8a,b shows the pressure distribution diagram of the linear structure, which shows that the pressure in the channel increased gradually from the outlet to the inlet. Figure 8c,d shows the pressure from the outlet to the inlet of the serpentine structure, which also increased gradually, but the pressure at the inlet of the serpentine structure was significantly greater than the pressure at the inlet of the linear structure (Figure 8e). Therefore, when injecting fluid into the channel, the serpentine structure at the entrance could prevent the fluid from flowing backward too fast and causing an unstable flow rate because of the high pressure; therefore, the serpentine structure was better than the linear structure, and using the serpentine structure can promote better mixing of reagents.

**Figure 9:**
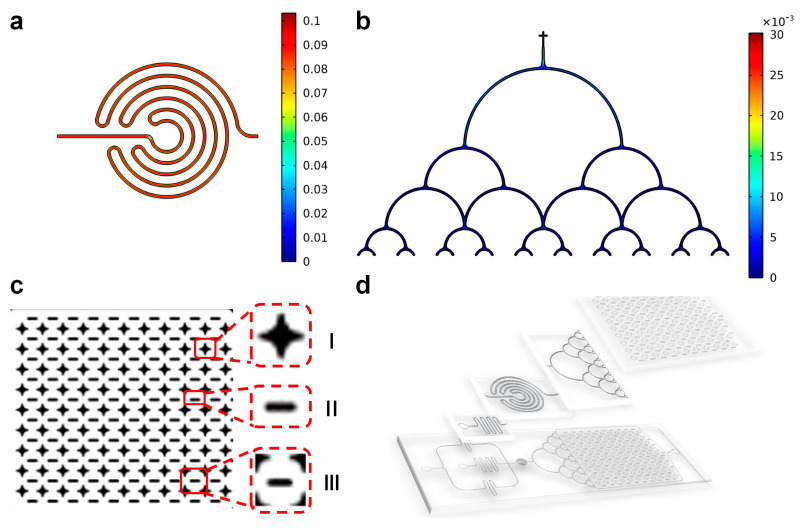
(**a**) Velocity distribution in microchannel with spiral structure of stable droplets; (**b**) tiled structure that allows the generated droplets to enter the collection chamber; (**c**) droplet collection chamber, small observation areas made by two types of support columns; (**d**) designed sketch of droplet microfluidic chip.

The spiral structure was designed to stabilize the generated droplets and mix the solution in the droplets. Figure 9a shows that the velocity distribution of the spiral structure was uniform, which was more conducive to droplets flowing steadily. Figure 9b shows that the velocity of the droplet from each outlet of the splitting structure was the same, so that the generated droplets could be evenly spread in the collection chamber. As shown in Figure 9c I–II, two types of support columns were designed to prevent the collection chamber from collapsing, and the combination of two different types of support columns could be regarded as the area shown in Figure 9c III. It was convenient for later detection, convenient for observation and counting, and improved the efficiency of analysis and detection. Figure 9d shows the effect diagram of the droplet microfluidic chip, which comprised an inlet, serpentine structure, droplet generation structure, spiral structure, splitting structure, and collection chamber.

**Figure 10:**
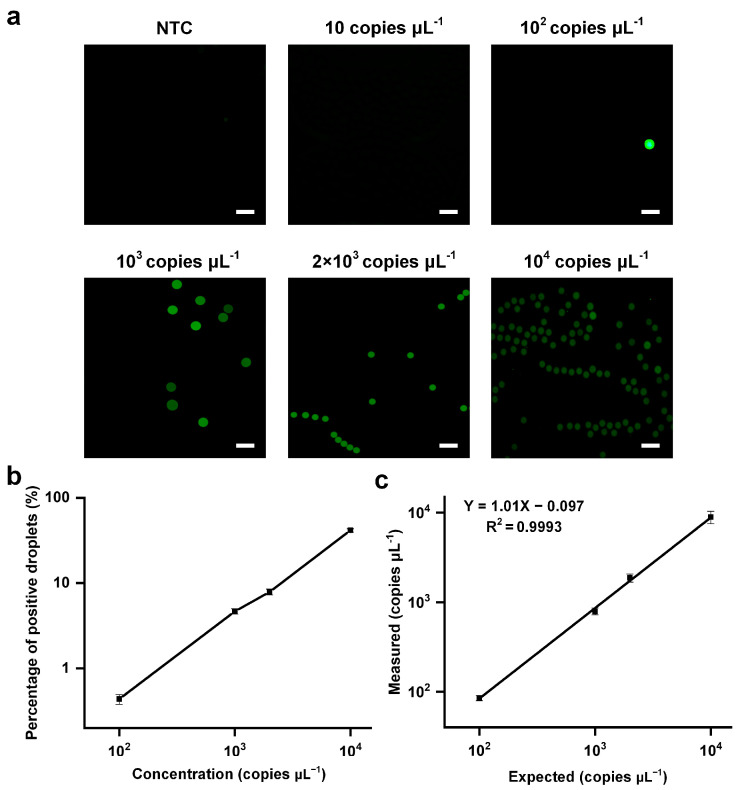
Digital-lamp detection SARS-CoV-2 results: (**a**) fluorescence diagrams of positive droplets after amplification with different sample concentrations; (**b**) probability of positive droplet number corresponding to different sample concentrations; (**c**) number of positive droplets corresponding to different sample concentrations (scale bar: 200 μm).

Finally, to study the quantitative analysis capability of droplet generation chip detection, we used the NTC and IVT samples with concentrations of 1 copies μL^−1^–10^5^ copies μL^−1^, mixed them with the LAMP mixture, generated 2 × 10^4^ droplets with an effective diameter of 10^3^ μm on the droplet generation platform, heated them in a water bath for 40 min, and finally analyzed them with a fluorescence microscope. Figure 10a shows the detection results of NTC and IVT samples with concentrations of 10 copies μL^−1^, 10^2^ copies μL^−1^, 10^3^ copies μL^−1^, 2 × 10^3^ copies μL^−1^, and 10^4^ copies μL^−1^, respectively. The results showed that with the increase of detection concentration, the percentage of positive droplets in the total number of droplets increased, and the number of positive droplets increased (Figure 10b,c). There was a strong linear relationship between the detected concentration and the input concentration Y = 1.01X − 0.097 (R^2^ = 0.9993). The lowest detection limit was 10^2^ copies μL^−1^ and, therefore, high-sensitivity detection of nucleic acids was realized.

## 4. Conclusions

Signal amplification using digital-LAMP offered extremely high sensitivity because the microfluidic droplet generation chip could treat each droplet as an independent LAMP reaction system. In early-stage experimentation, COMSOL Multiphysics was used to construct microfluidic structures with different functions for simulation. Through the comparison of the results, we chose the flow-focusing structure as the droplet generation structure and the serpentine structure as the fluid buffer structure. In addition, the spiral structure could save space and stabilize the droplets. The splitting droplet structure ensured that the droplets spread evenly in the collection chamber, which was convenient for statistical observation. The simulation results of different structural models by COMSOL Multiphysics can provide an important reference for the design of droplet microfluidic chips, save time, reduce costs, improve experimental efficiency, and provide a reference for other researchers when designing microfluidic chips at the same time. Digital-LAMP not only realized the high-sensitivity detection of nucleic acids but also reduced the requirement for experimental equipment to only an injection pump, fluorescence microscope, and water bath heating system. As shown in Table 2, we have summarized several works on digital-LAMP and compared their performances. In comparison, this paper provided a relatively complete work, from design to simulation to final detection. We designed several different structures and simulated their properties using COMSOL Multiphysics software. The simulation results were used to guide the design of the chip. The platform can meet the same detection requirements as other tools without requiring large confocal equipment. The lowest detection limit of this method was 10^2^ copies μL^−1^. All of these factors made this method very attractive for use in rural areas and laboratories with limited equipment. Additionally, the highly integrated chip could provide a detection platform and reference basis for the detection of other viruses.

## Figures and Tables

**Table 1:** Primer sequences used for RT-LAMP of the N gene in this study.

Primer	Sequence (5′ to 3′)
N-F3	TGG CTA CTA CCG AAG AGC T
N-B3	TGC AGC ATT GTT AGC AGG AT
N-FIP	TCT GGC CCA GTT CCT AGG TAG TCC AGA CGA ATT CGT GGTGG
N-BIP	AGA CGG CAT CAT ATG GGT TGC ACG GGT GCC AAT GTG ATCT
N-LF	GGA CTG AGA TCT TTC ATT TTA CCG T
N-LB	ACT GAG GGA GCC TTG AAT ACA

**Table 2:** The content of the study is compared.

Detection Method	Whether to Introduce the Chip Design	Is there any Reference for Simulation Results	LOD	References
Digital LAMP	No	No	25 copies μL^−1^	[20]
Digital LAMP	Yes	No	50 copies μL^−1^	[21]
Digital LAMP	Yes	No	10^−5^ fg μL^−1^	[22]
Digital LAMP	Yes	Yes	—	[23]
Digital LAMP	Yes	No	11 copies μL^−1^	[24]
DropCRISPR	Yes	No	102 cfu mL^−1^	[25]
Digital droplet RPA	Yes	No	100 copies μL^−1^	[26]
MF-LAMP	Yes	Yes	1 pg μL^−1^	[27]

## Data Availability

Not applicable.
